# Supplementary material for: The Construction and Exploration of a Comprehensive MicroRNA Centered Regulatory Network in Foxtail Millet (Setaria italica L.)
Source: Front Plant Sci. 2022 May 6;13:848474. doi: 10.3389/fpls.2022.848474 (PMC9121102; doi:10.3389/fpls.2022.848474)

T=Seita.1G300600.1_Q=Sit−miRN3020_S=1453

category=2_p=0.999999999999194

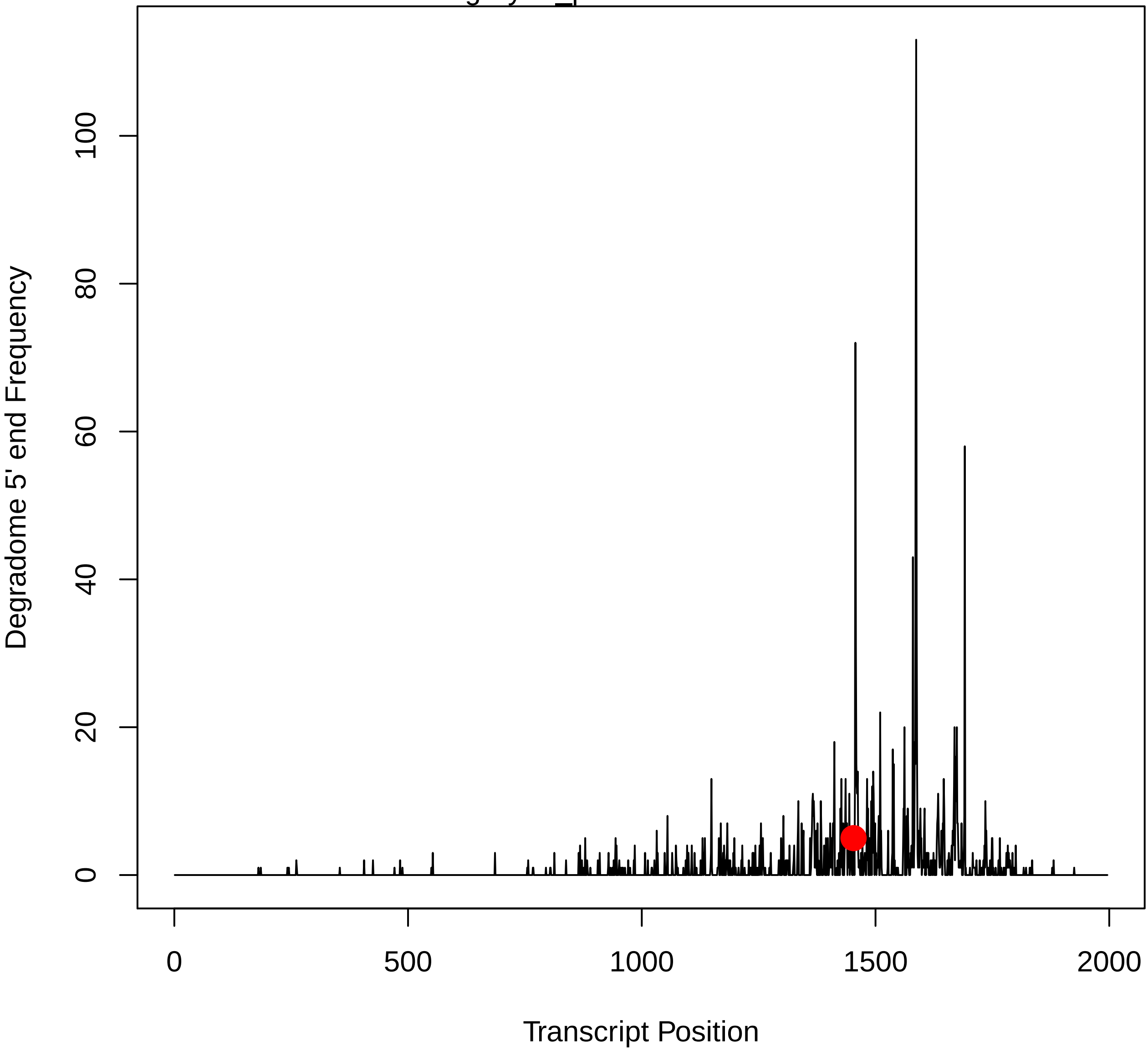

Supplement: Supplementary file 1 [file Data_Sheet_1.zip › Sit-miRN3020_Seita.1G300600.1_1453_TPlot.pdf]
